# FunGeneNet: a web tool to estimate enrichment of functional interactions in experimental gene sets

**DOI:** 10.1186/s12864-018-4474-7

**Published:** 2018-02-09

**Authors:** Evgeny S. Tiys, Timofey V. Ivanisenko, Pavel S. Demenkov, Vladimir A. Ivanisenko

**Affiliations:** 1 The Institute of Cytology and Genetics, The Siberian Branch of the Russian Academy of Sciences, Prospekt Lavrentyeva 10, 630090 Novosibirsk, Russia; 2 Laboratory of Computer Genomics, Novosibirsk State University, Pirogova Str. 2, 630090 Novosibirsk, Russia

**Keywords:** Gene set analysis, Node permutation, Random networks, Gene networks, Modular organization

## Abstract

**Background:**

Estimation of functional connectivity in gene sets derived from genome-wide or other biological experiments is one of the essential tasks of bioinformatics. A promising approach for solving this problem is to compare gene networks built using experimental gene sets with random networks. One of the resources that make such an analysis possible is CrossTalkZ, which uses the FunCoup database. However, existing methods, including CrossTalkZ, do not take into account individual types of interactions, such as protein/protein interactions, expression regulation, transport regulation, catalytic reactions, etc., but rather work with generalized types characterizing the existence of any connection between network members.

**Results:**

We developed the online tool FunGeneNet, which utilizes the ANDSystem and STRING to reconstruct gene networks using experimental gene sets and to estimate their difference from random networks. To compare the reconstructed networks with random ones, the node permutation algorithm implemented in CrossTalkZ was taken as a basis. To study the FunGeneNet applicability, the functional connectivity analysis of networks constructed for gene sets involved in the Gene Ontology biological processes was conducted. We showed that the method sensitivity exceeds 0.8 at a specificity of 0.95. We found that the significance level of the difference between gene networks of biological processes and random networks is determined by the type of connections considered between objects. At the same time, the highest reliability is achieved for the generalized form of connections that takes into account all the individual types of connections. By taking examples of the thyroid cancer networks and the apoptosis network, it is demonstrated that key participants in these processes are involved in the interactions of those types by which these networks differ from random ones.

**Conclusions:**

FunGeneNet is a web tool aimed at proving the functionality of networks in a wide range of sizes of experimental gene sets, both for different global networks and for different types of interactions. Using examples of thyroid cancer and apoptosis networks, we have shown that the links over-represented in the analyzed network in comparison with the random ones make possible a biological interpretation of the original gene/protein sets. The FunGeneNet web tool for assessment of the functional enrichment of networks is available at http://www-bionet.sscc.ru/fungenenet/.

**Electronic supplementary material:**

The online version of this article (10.1186/s12864-018-4474-7) contains supplementary material, which is available to authorized users.

## Background

At present, the reconstruction of molecular genetic networks (gene networks) is one of the most widely used approaches for studying the mechanisms of the functioning of complex biological processes. The use of this approach is often a necessary requirement for solving many problems in the field of biology, medicine, and pharmacology, among others [1–7].

Around the world, many databases containing molecular genetic networks describing metabolic processes, diseases, phenotypic traits, etc. have been developed – for example, KEGG PATHWAY [8], BioCyc [9], BioGRID [10] and IntAct [11].

There are systems that allow the reconstruction of gene networks for a given set of genes/proteins including FunCoup [12], STRING [13], Pathway Studio [14], Ingenuity Pathway Analysis [15], PINA [16], GeneMANIA [17] and ReactomeFIViz [18]. These systems use various information sources on interactions of molecular genetic objects, including scientific publications and factual databases. FunCoup is one such system containing more than 37 million interactions that include mRNA/protein co-expression, protein–protein interaction, similarity by phylogenetic profile, binding of shared transcription factors, sub-cellular co-localization and others. STRING is another example of such systems, containing information about protein–protein associations, information obtained from curated databases, predictions (gene neighborhood, gene fusions, gene co-occurrence), text-mining, co-expression, etc.

Earlier, we developed the ANDSystem, which has a wide range of tools for the reconstruction of associative gene networks [19]. The knowledge base of ANDSystem contains more than 14 million interactions between proteins, genes, metabolites, microRNAs, diseases, biological processes, etc. Information on interactions was extracted from PubMed abstracts using a text-mining method and was also extracted from various molecular genetic databases. Interactions were subdivided into physical interactions, catalytic reactions, chemical transformations, associations, regulation of expression, activity, transport/release, stability/degradation, etc. The ANDSystem was used to solve a wide range of tasks related to the reconstruction of gene networks – in particular, for the interpretation of data of proteomic experiments [20–22], the analysis of the tissue-specific effect of gene knockout [23], the analysis of the hepatitis C virus interaction [24, 25], the identification of genes susceptibility to tuberculosis [26] and analysis of molecular mechanisms of comorbidity of diseases [27, 28].

Another well-known approach to the study of functional linkages in gene sets is analysis of over-representation of the Gene Ontology (GO) biological processes, KEGG pathways and diseases. There are several computer tools aimed at facilitating this task, such as DAVID [29], BINGO [30], GO-function [31] and others. These programs are widely used to interpret the experimental sets of genes obtained in transcriptome analysis, genome-wide association studies, mass spectrometric experiments, etc. [22, 32–35]. However, such methods do not take into account a structure of the networks, which describe interactions between genes. Due to this, for the last ten years, several methods allowing to perform an analysis of gene networks were developed [36–39]. One such method is EnrichNet [37], which uses a random walk procedure for the estimation of the distance between experimentally obtained and predefined functional gene sets inside a network. Comparison of gene networks with random networks is an alternative approach for determining functional connectivity in experimental sets of genes/proteins [40–42]. In the work of McCormack et al. [43], a stand-alone tool, CrossTalkZ, was developed to assess the statistical significance of inter and intra-connectivity (crosstalk enrichment) between or within gene sets. CrossTalkZ uses the FunCoup database for the reconstruction of the gene networks, while random networks are generated by the permutations of all edges or nodes in a global network [12].

In this paper, we describe a web tool that allows evaluation of the functional relationship between genes using the STRING and ANDSystem databases, which differ from FunCoup by types of interactions between objects as well as information sources. Based on the analysis of the gene sets involved in GO biological processes, it is shown that the sensitivity of the method exceeds 0.8 at a specificity of 0.95 for both STRING and the ANDSystem. This study identified that the significance of the difference between gene networks of biological processes and random networks depends on the type of interactions (protein-protein interaction, co-expression, expression regulation, etc.). In particular, networks constructed for apoptosis (GO), including separate types of links, such as “activity and transport regulation”, “catalysis”, “co-expression” and “interaction”, were statistically significantly different from random networks. However, as a rule, the greatest reliability was observed for networks that included not individual types of links, but a general type of connection – that is, a type of connection in which two objects are considered to be connected if there is a link between them of any particular form. The FunGeneNet web tool allows users to upload a list of human gene/protein identifiers as an input. The output data is an associative gene network built either by the ANDSystem or STRING, as well as the evaluation of network functionality, expressed as the significance of the network enrichment with links of a given type. FunGeneNet is available at URL: http://www-bionet.sscc.ru/fungenenet/.

## Implementation

### FunGeneNet algorithm

In the first step, the network is automatically reconstructed for the input list of genes/proteins, using the ANDSystem or STRING base of knowledge. The networks used by FunGeneNet are subnetworks obtained from of the global ANDSystem or STRING networks. In the STRING networks, vertices correspond only to the proteins, linked by a generalized type of interaction. In the ANDSystem, genes and proteins are represented by separate objects, which can be linked by various types of interactions, including protein-protein interactions, protein-DNA interactions, regulation of gene expression, activity regulation, etc. In the next step, a filtration of the subnetwork by user-specified interaction type is performed. There are two operation modes in FunGeneNet. The first mode is applied when a user selects “all types” for the interaction. In this case, all interactions presented in the FunGeneNet network are considered as a generalized type of interaction. The second mode is used when a user selects a specified type of interaction (for example, “activity regulation and transport”, “catalysis”, etc.). In this case, the system employs only interactions of the specified type, while any others are removed from the network. It should be noted that in the case of STRING, only the generalized interactions are used.

The method for assessing the functional enrichment consists of comparing the number of links between the analyzed and random networks. For this purpose, the connectivity of 100 random networks is calculated and the parameters of the normal distribution are evaluated for this sample to use a one-sided single-sample t-test (pnorm function of R language). In the absence of connections in both the analyzed and all random networks (edgeless networks), the *p*-value is taken to be 0.5, since in the case of a small non-zero number of edges in the sample of random networks, the p-value for an edgeless network is close to 0.5.

For the reconstruction of the random networks we used the node permutation approach proposed in [44]. The main difference of our algorithm is that labels of vertices were swapped in the global network, not in the local one. Other randomization methods were not considered because they are significantly inferior in performance to the method of node permutation and do not yield a significant gain in accuracy [43]. Performance in this study was critical because FunGeneNet is a web-application.

Random networks were built according to the following rules: (1) For each protein of the analysed set, the vertex degree in the global network was counted and the set of proteins of the global network with the same vertex degree was determined; (2) One protein was randomly selected from this set, which served as the starting vertex for the reconstruction of the random network; (3) The network reconstruction for the starting vertices was performed as for the network being analyzed.

Thus, each random network contained the same number and type of vertices as the original network, and the link types were also the same, while the number of links in random networks and the original were different due to permutations.

**Fig. 1:**
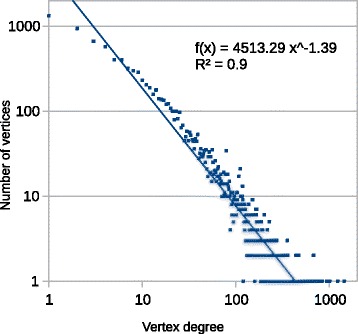
Distribution of vertices in the ANDSystem global human gene network by vertex degree. Black colour identifies the trend line of the number of vertices f(x), where x is the vertex degree. R^2^ is the coefficient of determination

Restriction (1) on the degrees of selectable vertices in the global network is aimed at reducing the study bias described by Jensen et al. [45] as a tendency to study, in various aspects, primarily well-studied molecules. In this connection, we assume that vertices with relatively large degrees (hubs) accumulate more false-positive interactions than vertices with lower degrees. As can be seen from Fig. 1, the vertex degrees in the global gene network can be roughly described by a power law with the coefficient γ = 1.39. Therefore, the probability of choosing at random a vertex with a small degree is significantly higher than the probability of choosing a hub. Thus, if in the studied group of genes/proteins the hubs predominate for some reason, then such a network is likely to be more connected than the networks with randomly selected genes. The presence of well-studied genes in the analyzed sample can lead to a systematic error in random sampling, which was also noted in other works [40, 43].

### FunGeneNet input data

A list of protein IDs for the following databases is supplied to the input: UniProt, Ensemble. The program also understands NCBI gene identifiers. In a case where genes are fed to the input of the tool, the list of encoded proteins is first determined, and then the reconstruction is performed. The user has the opportunity to select the STRING system or the ANDSystem, through which the gene network will be reconstructed. In the case of using STRING, the user can select one of the standard thresholds for the presence of a connection in the global network: 150, 400, 700 and 900. In the case of the ANDSystem, the user can select the type of interaction from the list (activity and transport regulation, catalysis, coexpression, expression regulation, interaction and all types).

### FunGeneNet output data

The FunGeneNet output is a file containing an interactive network in ANDSystem/tab-delimited format and the t-test *p*-value, which characterizes the difference between the analysed network and random networks. The given t-test *p*-value assumes the normal distribution of the number of links in random networks and can be biased from the true probability values. Therefore, in addition to the network being analyzed, the ROC curve *p*-value is calculated as the proportion of negative sample networks having a t-test *p*-value less than or equal to that for the network being analyzed (coords function of the pROC package of R language).

### Accuracy estimation of the FunGeneNet method

To analyze the accuracy of the FunGeneNet method, we applied the ROC analysis technique [46]. Networks constructed for GO biological processes were considered as a positive sample. Information on the involvement of proteins in the processes was taken from the UniProt-GOA database (Submission date: 3/16/2016) [47]. GO networks were divided into two groups according to the number of proteins. The first group included processes for which 2 to 50 proteins were annotated, and the second group included processes with more than 50 proteins according to UniProt-GOA (Additional file 1: Table S1).

As a negative sample of networks, four types of random networks were used, for which it was assumed that they include functionally unrelated genes. Networks of the first type (simply random) were constructed by randomly selecting proteins from the whole set of human proteins, each of which had at least one connection in the global ANDSystem network. This restriction, to exclude proteins not participating in the formation of the global network, is also applied to other types of random networks. To build networks of the second type (well-studied), a random selection was made from proteins, mentioned in at least 50 PubMed publications. Thus, this group was represented by the relatively well-studied proteins. This group was created in order to take into account the possible FunGeneNet misclassification bias introduced by the level of scrutiny of proteins [45]. Networks of the third type (GO-based) were built using a random selection of proteins from a variety of proteins annotated in the GOA database (Additional file 2: Table S2). The reconstruction of these networks was carried out in such a way that one network did not contain the proteins involved in the same biological process. Networks of the fourth type (identical degree distribution [IDD]) were constructed with a restriction on the vertex degrees, so that each set of proteins from the positive sample corresponded to a set of the negative sample. The selection procedure consisted of three steps: (1) the vertex degree in the global network is determined for each protein of a positive sample, (2) the list of all proteins with the same degree as for a particular protein of a positive sample is extracted from the global network, (3) the starting protein for IDD network reconstruction is selected at random from this list. This method of reconstruction guaranteed equal vertex degree distributions in positive and negative samples. When considering characteristics of FunGeneNet – depending on the size and completeness of the networks, the STRING score, and the t-test/permutation option – networks of the type “simply random” (Additional file 2: Table S2) were used.

To construct the ROC curves, the number of random networks in a negative sample, as well as the distribution of the number of proteins in the random networks were specified to be equal to those in the positive sample. The same positive and negative samples of proteins were used to reconstruct networks for the ANDSystem and STRING (version 9.1).

The ROC curve classifier score was taken to be equal to 1 − *p*-value, where p-value characterized the statistical significance of the differences between the analysed networks and random networks, given out in the output data of the program. The area under the ROC curve (AUC) was calculated using the “roc” function of the pROC package of R language. As the “roc” argument “auc”, a “predictor” vector consisting of values of 1 − *p*-value for functional and random networks was fed. The argument “response” was a vector, with the coordinate values equal to 1 for functional networks and 0 for random networks.

To analyze the performance of the method depending on the type of interactions, the ANDSystem types were combined into larger types: (1) “activity and transport regulation”, which included the following types of interactions: “activity downregulation”, “activity regulation”, “activity upregulation”, and “transport regulation”; (2) “catalysis”, including “catalyze”, “cleavage”, “degradation downregulation”, “degradation regulation”, and “degradation upregulation”; (3) “coexpression”, which was taken as a separate type; (4) “expression regulation”, consisting of “up-”, “down-”, and “expression regulation” itself; (5) “interaction”, which was taken as a separate type; and (6) “all types”, including all of the above types, as well as the type “expression” and the type “association”.

To estimate how the completeness of genes of the studied process, presented in the experimental set of genes, would affect the obtained results, the following analysis was performed. At the first step, all GO biological processes were divided into five main groups according to the number of genes involved in each process: (1) processes, involving 10 genes; (2) from 20 to 22 genes; (3) from 40 to 50 genes; (4) from 100 to 200 genes; (5) from 400 to 1000 genes. Next, for each process, 10 genes were randomly selected from its entire set of genes. Thus, the completeness for the first group was 100% (the experimental set contained all genes of the process), for the second it was 45–50%, for the third it was 5–10%, etc. The selected lists of proteins are given in Additional file 1: Table S1. At the next step, an ROC curve was constructed for each range of the completeness.

The significance of the difference in the AUC of the ROC curves was estimated using the two-sided unpaired DeLong’s test, through the roc.test function of R language.

The p.adjust function of R language was used for the Benjamini Hochberg multiple testing correction.

## Results

### Method assessment

**Fig. 2:**
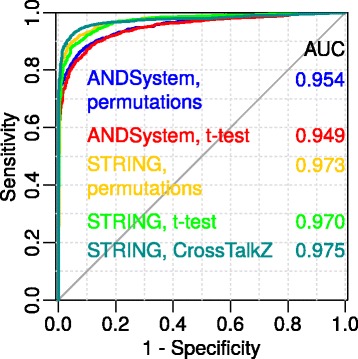
Method accuracy for t-test. GO sets including from 11 to 50 proteins were used as a positive control. “Simply random” sample was used as a negative control

We consider two method variants, based on 1000 permutations as well as the t-test, using parameters of normal distribution estimated from 100 permutations. To assess any decrease in accuracy in the case of using the t-test instead of permutations, we build ROC curves for these variants (Fig. 2). Figure 2 shows that the AUC for these variants is nearly the same for both the ANDSystem and STRING. Due to this, and based on the fact that the method variant using a t-test reduces the number of calculations by approximately 10 times, below we show ROC curves constructed by the method based on the t-test.

**Fig. 3:**
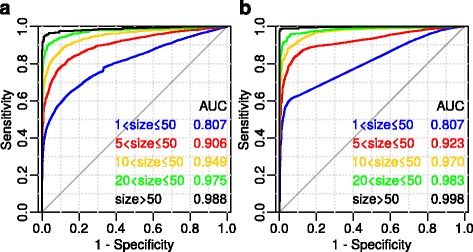
Dependence of the method accuracy on the size of the networks for ANDSystem (**a**) and STRING (**b**). The size of the network (designated as “size”) was defined as the number of proteins annotated with the GO biological process. As a negative sample, the sample “simply random” was taken (see methods). The STRING score was used by default (= 400)

An interesting question about the FunGeneNet applicability is the dependence of the quality of the functional/non-functional network classification on the size of the gene set. Figure 3 shows that FunGeneNet performs non-random classification even in cases of small network sizes.

**Fig. 4:**
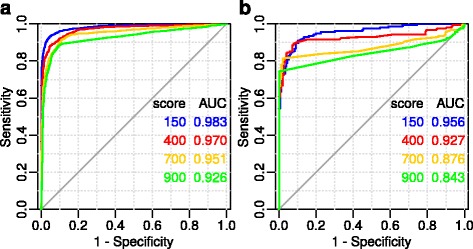
ROC-curves for different values of STRING score for networks of size > 10 and ≤ 50 (**a**) and for networks of size = 10 (**b**). In all variants, as a negative control, all genes with at least one bond in the global network with a score above 400 were taken. Edgeless networks correspond to the linear sections of the ROC curve. Linear segments are due to a fairly large proportion of edgeless networks with the same *p*-values in positive and negative controls

Interactions between the genes contained in the global network have a different degree of reliability. Therefore, in the STRING system, a special score is used, which describes the weight of interactions. The STRING score is the threshold for eliminating noise information. Increasing the score for STRING networks can reduce the share of false interactions and decrease the completeness of networks. For this reason, a decision was made to check how the accuracy of FunGeneNet depends on the STRING score. Figure 4 shows the ROC curves for the standard values of the STRING score.

**Fig. 5:**
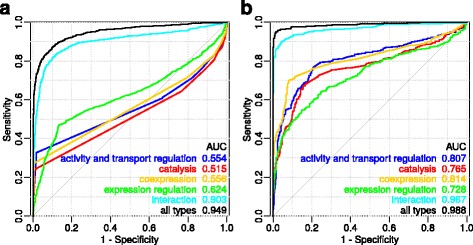
ROC-curves for different types of interactions for ANDSystem networks of size from 11 to 50 genes (**a**) and over 50 (**b**)

The use of ANDSystem networks in FunGeneNet allows analysis of different types of interactions, including all types (generalized type), activity and transport regulation, catalysis, co-expression, expression regulation, and interaction. Figure 5 shows the ROC curves for the different interaction types from the ANDSystem according to the different network sizes.

**Fig. 6:**
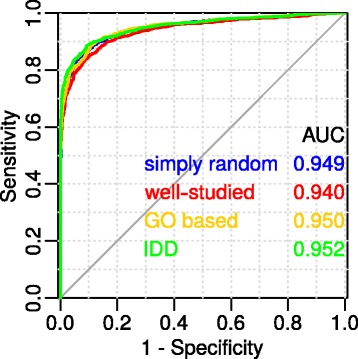
The quality of the method for different models of negative control. The size of the networks used in the comparison lies in the range: 10 < size ≤ 50

Another important issue to assess the quality of the method is the appropriate sampling of non-functional networks. We proposed four models of non-functional networks: “simply random” — random selection of a set of proteins, from having at least one connection in the global network; “well-studied” — the choice is the same as in “simply random”, but from proteins found in more than 50 publications; “GO based” — random selection is made from GOA, so that all the proteins in the sample do not have common GO biological processes in the direct GOA annotation; “the same degree of distribution” (IDD) — with this choice of negative control, the vertex degree distributions (vertex degrees are counted using the global network) in negative protein samples are exactly the same for those of positive samples. Figure 6 illustrates the ROC curves for the ANDSystem for various models of negative control.

**Fig. 7:**
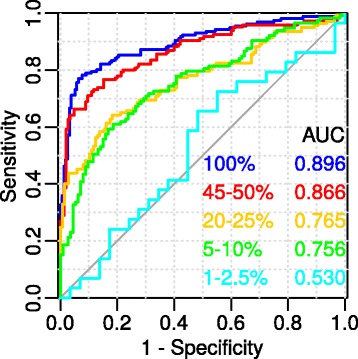
ROC-curves for different completeness of networks. The percentages indicate the completeness of the network, which was calculated as the ratio of the random sample size of the GO biological process, chosen to construct the ROC curve to the full size of this process (See methods). Each of the considered networks includes 10 genes

Since, in an experimental gene/protein set that can be analyzed with the help of FunGeneNet, for some reason only a small part of the biological process under investigation may appear, we explored how much the accuracy of the method depends on the completeness of the data on the observed process. Figure 7 shows the ROC curves for different portions of GO biological processes for which the network is built. It can be observed from the figure that, as expected, with a decrease in the proportion of proteins over which the network is built, the area under the ROC curve decreases. For protein sets composed of 5–10% of all proteins assigned to the GO biological process, the classification is weaker, but not yet random, and for sets of 1–2.5%, it is close to random.

### Thyroid cancer network

Papillary thyroid cancer is the most common form of thyroid cancer [48]. In the dbDEPC database, we identified data from three experiments on papillary thyroid cancer: EXP00039 (E39), EXP00050 (E50) and EXP00051 (E51). E39 contained a list of 30 differentially expressed proteins [49]. E50 and E51 were conducted within the same work and gave an identical list of 16 proteins for two different variants of cancer cell types [50]. At the intersection between the E39 and E50 lists, there were five proteins: ANXA1, Beta-actin, Moesin, FTL and Galectin-3.

Using FunGeneNet, we reconstructed networks for E39 (Additional file 3) and E50 (Additional file 4), as well as for intersection (Additional file 5) and union (Additional file 6) of the protein lists. The results of comparing networks E39 and E50 with random networks are listed in Table 2.

### Apoptosis network

As an example, we considered a functional network formed by genes/proteins participating in the GO apoptotic process [GO: 0006915]. Apoptosis is known to be necessary for the normal development and functioning of the organism and is also of key importance in mechanisms of many diseases, such as neurodegenerative and cancer diseases [51–53]. A wide range of interactions is involved in this process, including the protein–protein interaction and regulatory links that determine the regulation of gene expression, as well as the regulation of protein activity and transport, etc. The identification of the significance of different connection types in the gene network of the apoptotic process can help to better understand the mechanisms of functioning and the role of participants of this complex biological process.

The protein list of apoptosis according to UniProt-GOA included 593 proteins (Additional file 7: Table S4). The network included 591 proteins, 585 genes and 12,529 interactions (Additional file 8: apoptotic process.andz).

FunGeneNet established the apoptosis network as functionally enriched by the types of “activity and transport regulation” (*p*-value = 3.95e-09), “catalysis” (*p*-value = 3.06e-06), “coexpression” (p-value = 3.09e-02), “interaction” (p-value = 3.24e-76) and “all types” (ANDSystem p-value = 1.46e-30, STRING p-value = 0). All networks for these types of links generally correspond to the power law of vertex degree distribution (Additional file 7: Table S4). This means that a small fraction of vertices aggregates most of the connections and these vertices can be of considerable interest.

## Discussion

### Method assessment

From Fig. 2, we can see that CrossTalkZ as compared to FunGeneNet showed a slightly higher AUC value. Nevertheless, due to its low performance, this program was not used in our system. In particular, this is due to the permutation of all the vertex names of the global network, while our algorithm is based on the permutation of only those nodes of the global network that are relevant to the analyzed one.

An increase in the number of correctly predicted functional networks with an increase in their size (Fig. 3) may be explained by an information noise decrease in the networks. The noise is a proportion of false positive links in the analyzed network. To detect a significantly higher number of links in the functional network compared with the random ones, it is required that the signal/noise ratio in the functional network be greater than that in the random networks. Another reason for the slight difference of small networks from random ones may be their incompleteness in comparison to large networks. This is because the meaningful part of the small networks has only been studied recently and not all genes involved and links between them have been revealed.

From Fig. 3 it is seen that for α = 1 − specificity = 0.05 for networks of a size greater than 10 and not greater than 50, the sensitivity exceeds 0.8 for both the ANDSystem and STRING. Further, we use this group of GO biological processes to examine the behaviour of the ROC curves in various conditions.

It is interesting that for the networks with sizes from 2 to 50 genes in Fig. 3a, a sensitivity jump by 0.02 is detected in the range of α [0.33, 0.34]. This jump corresponds to edgeless networks of positive and negative samples, for which all the random networks turned out to be edgeless. This jump is due to there being fewer networks in the negative sample (29 from 3458 edgeless) compared to the positive sample (115 from 1211). This fact is most likely connected with the difference in the distribution of vertex degrees in functional and non-functional networks. This question deserves a separate consideration with the goal of constructing, based on the vertex degrees in the global gene network, a method for classifying functional and non-functional gene sets. For GO biological processes including more than 10 genes, there are only six such edgeless networks. Thus, the contribution of edgeless networks is insignificant and the jump on the chart is invisible.

As can be seen in Fig. 4a, the highest AUC is observed for a STRING score of 150, and the smallest for a score of 900. This indicates that the method works better in complete but noisy networks, compared to networks with a small fraction of false positive interactions, with a high proportion underpredicted. Figure 4b shows the same pattern on networks with a fixed size of 10. This dependence is even more pronounced, apparently because of the small size of the networks and the absence of its variability. In a small-sized network, there are only a few well-established links, and that is why long linear segments corresponding to edgeless networks for scores of 700 and 900 appeared in Fig. 4b. It can be shown that the greater the score, the longer the linear sections and the more edgeless networks.

It appeared that with the increase of network size, the quality of classification grows (Fig. 5). The most accurate among the considered types of interactions appeared to be “all types”. Such a result was expected, since the consideration of only a specific type of interaction (for example, only transport regulation) leads to information loss, while the use of a generalized type avoids this [54, 55]. It should be noted that the quality of classification for some interaction types (for example, “catalysis” or “activity and transport regulation”) can be explained by the smaller number of genes involved in the GO biological process linked by such types, which, in particular, may cause the appearance of edgeless networks.

From 1625 processes involving from 11 to 50 proteins, 1507 differed in at least one type of connection (Additional file 9: Table S3). For each type of link, there were processes that differed only by this type of link (Table 1). This means that, having examined the differences from random networks by the mixed type of links (“all types”), we under-predict some functional networks. We tested if there are any common properties for networks that differ in a certain type. It turned out that for the “expression regulation” type among 21 significant GO biological processes that do not differ from random networks by “all types”, a group of 10 processes are distinguished, which are related to cell proliferation and the cell cycle: negative regulation of B-cell proliferation, positive regulation of phosphorylation, negative regulation of cyclin-dependent protein serine/threonine kinase activity, negative regulation of proteasomal ubiquitin-dependent protein catabolic process, positive regulation of cell cycle, negative regulation of organ growth, homeostasis of number of cells, positive regulation of cellular component movement, regulation of protein kinase B signalling and regulation of actin cytoskeleton reorganization. In addition to this group, it is possible to identify a more specific group of B-cell proliferation (negative regulation of B-cell proliferation, cellular response to interleukin-4, cellular response to interleukin-6). IL-4 (BSF-1) and IL-6 stimulate B-cell proliferation [56, 57]. Another group of processes for the “expression regulation” type refers to cell differentiation (trophectodermal cell differentiation, monocyte differentiation, and endothelial cell differentiation). Thus, it can be assumed that the networks of some functionally related GO biological processes are more different from random networks by a certain type of interaction compared with networks for other GO biological processes.

**Table 1:** The distribution of the number of networks of GO biological processes statistically significantly different from random ones for different interaction types

Interaction type	Number of networks different by at least two interaction types	Number of networks different by only the interaction type	Number of networks not different by “all types”
Activity and transport regulation	392 (0.26)	7 (0.005)	10 (0.007)
Catalysis	293 (0.194)	3 (0.002)	8 (0.005)
Coexpression	424 (0.281)	5 (0.003)	17 (0.011)
Expression regulation	500 (0.332)	15 (0.01)	21 (0.014)
Interaction	1250 (0.829)	64 (0.042)	77 (0.051)
All types	1395 (0.926)	83 (0.055)	–

Values in parentheses are the fraction of the number of networks different by at least one type

The most represented type of links, not counting “all types”, was the “interaction” type. Of 1507 significant networks, 1250 differed by this type. However, if we exclude processes that are significant by the “all types”, then only 77 networks will remain. Increased representation of the interaction type in comparison with other types of interactions can be explained by the appearance of high-performance methods, such as mass spectrometry [58] and yeast two-hybrid analysis [59].

It can be demonstrated from the Fig. 6 that the AUC varies insignificantly with different models of negative control (*p*-value = 0.105 for comparison of “simply random” and “well-studied”). On the one hand, this shows that imposing a strict vertex limit on IDD does not turn random networks into functional ones, which could be expected, since the pool of vertices for random selection is greatly reduced. On the other hand, the proximity of “simply random”, “GO-based” and “well-studied” curves shows that the proposed increased examination of GOA-annotated genes, compared to random genes, does not significantly affect the quality of the method.

Since it is difficult to determine what is really a non-functional network, we consider random networks as non-functional networks. It is possible that of all the reconstructed random networks, some are functional networks, which can underestimate the sensitivity and specificity, because among these random functional networks there may be those with a connectivity that is higher than the connectivity of the analyzed functional networks. Perhaps, in the presence of such a phenomenon, the addition of a restriction on the vertex degrees in a random network (as in the IDD variant) may lead to an increase in the proportion of such false positive networks in the negative sample and a greater underestimation of the method accuracy. However, as can be seen from Fig. 6, this understatement does not occur. In addition, we showed that for networks larger than 10, the method works well enough. So, even with some portion of the functional networks among random ones, random networks are an acceptable model of negative control.

The result of the dependency analysis between accuracy of the method and completeness of the data on the observed process (Fig. 7) is important for choosing a strategy for analyzing experimental sets of genes/proteins using FunGeneNet, as well as other methods based on the analysis of gene networks constructed from experimental gene sets. The absence of the significant differences from random networks may be related to incompleteness of experimental gene sets with respect to the real number of genes involved in the studied biological process. By taking into account this fact, experiments can be adjusted. Another way to solve this problem can be the extension of an experimental set of genes by gene-prioritization methods [60]. In particular, our analysis of the different levels of completeness of experimental gene sets on the example of GO biological processes showed that in cases where an experimental set of genes was less than 2.5% of the total number of genes of the target process, the absence of significance of functional connectivity in gene sets can be expected.

### Thyroid cancer network

We were interested in identifying which genes/proteins in the Papillary thyroid cancer networks and their connections contribute to distinction from random networks (Table 2). The most important was the combined network of E50 and E39, which differed from random networks by the combined type “all types”, and the E50 network, which differed in the type of “catalysis”. Interestingly, the latter difference was due to the presence of two catalytic bonds in the protein transthyretin (TTR), which is a carrier of thyroid hormones. The involvement of TTR in thyroid cancer is consistent with the previously advanced hypothesis of an increased risk of thyroid cancer in the presence of particularly polybrominated diphenyl ethers (PBDEs), metabolites of which compete with thyroid hormones for binding to TTR [61]. Since TTR has catalytic activity [62], it can be assumed that PBDEs, through binding to TTR, change its catalytic activity. Interestingly, the second catalytic TTR link in the analyzed network is aimed at its cleavage by oncogene DJ-1, which, in an unknown way, regulates the phosphatidylinositol-3 kinase signalling pathway through the tumour suppressor PTEN [63]. Mutations in the PTEN gene lead to syndromes accompanied by cancer in various tissues, including the thyroid gland [63]. Thus, it can be assumed that TTR mediates the regulation of PTEN by DJ-1 protein.

**Table 2:** Enrichment by various types of functional interactions in groups of differentially expressed proteins in thyroid cancer (dbDEPC) estimated with FunGeneNet

Experiment	ANDSystem interaction type	*p*-value	BH corrected^a^* p*-value
E39	all types	3.83E-03 (1.47e-17)	1.84E-02
E39	activity and transport regulation	1.58E-01	3.45E-01
E39	catalysis	6.56E-01	7.50E-01
E39	coexpression	6.08E-01	7.50E-01
E39	expression regulation	7.27E-01	7.93E-01
E39	interaction	4.62E-01	7.06E-01
E50	all types	2.02E-02 (6.31e-05)	8.08E-02
E50	activity and transport regulation	1.09E-01	2.91E-01
E50	catalysis	1.45E-05	**1.74E-04**
E50	coexpression	5.00E-01	7.06E-01
E50	expression regulation	6.56E-01	7.50E-01
E50	interaction	6.58E-02	1.97E-01
E50 ∩ E39	all types	4.36E-01 (3.52E-03)	7.06E-01
E50 ∩ E39	activity and transport regulation	1.35E-01	3.24E-01
E50 ∩ E39	catalysis	5.00E-01	7.06E-01
E50 ∩ E39	coexpression	5.00E-01	7.06E-01
E50 ∩ E39	expression regulation	5.69E-01	7.50E-01
E50 ∩ E39	interaction	7.84E-01	8.18E-01
E50 ∪ E39	all types	6.42E-06 (3.48e-29)	**1.54E-04**
E50 ∪ E39	activity and transport regulation	4.03E-01	7.06E-01
E50 ∪ E39	catalysis	1.11E-04	**8.88E-04**
E50 ∪ E39	coexpression	1.19E-03	7.14E-03
E50 ∪ E39	expression regulation	8.89E-01	8.89E-01
E50 ∪ E39	interaction	4.71E-02	1.61E-01

*p* < 0.001 are highlighted in bold

STRING p-values are given in parentheses

^a^Benjamini-Hochberg correction, see methods

### Apoptosis network

An analysis of the gene network of GO apoptotic process [GO: 0006915] has revealed, that of the 90 transport regulation links, 30 links regulating the release of cytochrome c attract attention. This is consistent with the key role of cytochrome c in the mitochondria-dependent pathway of apoptosis [64]. The second leader by the number of “regulation of transport” connections is BAX protein. This protein aggregates 12 such bonds, of which nine show the influence of other proteins on BAX translocation from the cytosol into the mitochondria. This translocation is also the central event in the mechanism of apoptosis [65]. Among the links regulating activity, the maximum degrees of the vertices are for NFKB1 and P53. Among the 43 bonds, NFKB1 37 is directed at regulating the activity of this protein. As is known, this protein initiates apoptosis in order to suppress the development of tumours [66]. Of the 39 links of p53, 24 are directed to its regulation, this is consistent with p53’s key role in triggering apoptosis due to DNA damage, oncogenes expression and the effects of other factors [67]. For the “catalysis” type links, the participants with the highest degrees of vertices were the anti-apoptotic kinase AKT1, the proapoptotic CASP3, and the apoptosis-inducing p53. Among the 19 links of AKT1, 12 are phosphorylation of apoptotic proteins with AKT1. Of the 30 CASP3 links, 17 are cleavage of apoptotic proteins by CASP3. Of the 35 bonds of p53, 13 are responsible for regulation of its stability. For the co-expression network, a connected component was found containing four cellular receptors (CD2, IL2RA, TNFRSF18, and PRAME), kinase CDK11B and keratin KRT20. The maximum number of links in the co-expression network was three and was observed as adjacent to the CD2 protein. In the “interaction” network, Polyubiquitin-C (373 bonds), P53 (101 bonds) and CASP3 (67 bonds) were the leading proteins (Additional file 7: Table S4). When protein is ranked according to the specificity rate (SR = [number of connections in this network] / [number of connections in the global network]), the proteasome proteins PSMA8 (SR = 0.929) and PSMB6 (SR = 0.816) are leaders, as well as HIPPI protein (SR = 0.750), inducing apoptosis through activation of caspase-8 expression [68].

Thus, on the basis of analysis of different types of interactions in the gene network, describing the GO process of apoptosis, the connected components were identified, i.e. sets of genes involved in over-represented interactions. It appeared that these components include genes that are key for apoptosis.

### Network modularity

The difference between functional networks and random ones is in good agreement with the principle of modular organization of biological systems, which Hartwell et al. (1999) brought into focus [69]. According to their definition, the module is part of a biological system that has a function that can be separated from the function of other such subsystems. The reflection of the principle of the modular organization at the level of gene networks is that the genes belonging to one module are closely located in the network [70, 71]. The work carried out by Ames (2013) [54] showed that the cohesive sub-graphs of global networks constructed from experimental data of different types overlap significantly with each other and with GO. Furthermore, the combination of these networks increases the coverage of GO. Based on the network modularity in the studies of Dutkowski et al. [72], Gligorijevi’c et al. [55] and Kramer et al. [73], gene ontologies were constructed exclusively based on network topology. Such topologies have shown a significant intersection with the existing topology of GO. Thus, the difference between functional networks and those that are random in terms of the number of connections is in agreement with the modular principle of network organization. For example, FunGeneNet showed the significance of the functional connectivity of the set of genes involved in histone deubiquitination [GO:0016578] as being equal to 6.48e-21, calculated by the ANDSystem, which can be a functional module (NeXO:8805) according to the NeXO ontology [72].

## Conclusions

At present, using experimental transcriptomic, genomic and proteomic technologies, large arrays of experimental gene sets are generated. Such approaches are widely used to study medical-biological problems related to phenotypic traits, diseases, pathological conditions, etc. Reconstruction and analysis of gene networks that describe the functional interactions between genes in experimental sets of genes is a promising approach for the interpretation of omics data. FunGeneNet is dedicated to the analysis of the functional connectivity in experimental gene sets and identification of the most important links between genes from these sets, including the physical protein–protein interactions, protein–DNA interactions, and regulatory links such as regulation of expression, activity, etc. Reconstruction of gene networks for analyzed gene sets in FunGeneNet is carried out automatically using STRING and the ANDSystem.

The application of FunGeneNet to the analysis of gene sets involved in Gene Ontology biological processes has shown the statistical significance of the difference of networks reconstructed for these processes from random networks, which is in good agreement with the notion that functionally related genes participate in common biological processes. Sensitivity of our method exceeds 0.8, while specificity is 0.95.

The main feature of the method implemented in FunGeneNet is that it allows consideration of specific types of molecular-genetics interactions. An analysis of the connection types showed that the difference of GO biological processes from random networks depends on the types of interactions represented in them. Thus, genes involved in such processes can play an important functional role in analyzed processes. In particular, the analysis of a set of genes involved in apoptosis showed that such genes as NFKB1, P53, AKT1, CASP3 and HIPPI possess significant links, which is in good agreement with the literature data. Analysis of the gene sets associated with thyroid cancer taken from the dbDEPC database showed that these genes are significantly functionally related, and also suggests the molecular mechanisms of the role of genes involved in significant catalytic reactions.

An analysis of the gene sets associated with thyroid cancer taken from the dbDEPC database showed that these genes are significantly functionally related, and also allowed to suggest molecular mechanisms of the role of genes involved in significant catalytic reactions.

## Availability and requirements

**Project name:** FunGeneNet.

**Project home page:** http://www-bionet.sscc.ru/fungenenet

**Operating system:** platform independent.

**Programming language:** PHP, Python.

**Other requirements:** Any browser with HTML5 support.

**License:** GPL-3.

**Any restrictions to use by non-academics:** none

## Additional files

Additional file 1: Table S1. Positive protein samples. (XLSX 1846 kb)

Additional file 2: Table S2. Negative protein samples. (XLSX 2044 kb)

Additional file 3: The network for differetially expressed proteins for EXP00039 experiment. The file can be opened with the ANDSystem tool (http://pbiosoft.com/en/andsystem/andsystem-free). (ANDZ 83 kb)

Additional file 4: The network for differetially expressed proteins for EXP00050 experiment. The file can be opened with the ANDSystem tool (http://pbiosoft.com/en/andsystem/andsystem-free). (ANDZ 61 kb)

Additional file 5: The network for differetially expressed proteins for both EXP0039 and EXP00050 experiments. The file can be opened with the ANDSystem tool (http://pbiosoft.com/en/andsystem/andsystem-free). (ANDZ 21 kb)

Additional file 6 The network for differetially expressed proteins for EXP00039 and/or EXP00050 experiment. The file can be opened with the ANDSystem tool (http://pbiosoft.com/en/andsystem/andsystem-free). (ANDZ 123 kb)

Additional file 7: Table S4. Characteristics of apoptosis network. (XLSX 55 kb)

Additional file 8: The network reconstructed for the GO term ‘apoptotic process’. The file can be opened with the ANDSystem tool (http://pbiosoft.com/en/andsystem/andsystem-free). (ANDZ 3743 kb)

Additional file 9: Table S3. FunGeneNet classification results for different interaction types. (XLSX 254 kb)

## Abbreviations

AUC: Area under the ROC curve
E39: EXP00039
E50: EXP00050
E51: EXP00051
GO: Gene Ontology
IDD: Identical degree distribution
PBDEs: Particularly polybrominated diphenyl ethers
SR: Specificity rate
TTR: Transthyretin

## References

[1] Greenham K, McClung CR (2015). Integrating circadian dynamics with physiological processes in plants. Nat Rev Genet.

[2] Le Novère N (2015). Quantitative and logic modelling of molecular and gene networks. Nat Rev Genet.

[3] Mitra K, Carvunis A-R, Ramesh SK, Ideker T (2013). Integrative approaches for finding modular structure in biological networks. Nat Rev Genet.

[4] Peterson EJ, Ma S, Sherman DR, Baliga NS (2016). Network analysis identifies rv0324 and rv0880 as regulators of bedaquiline tolerance in mycobacterium tuberculosis. Nat Microbiol.

[5] Prathipati P, Mizuguchi K (2016). Systems biology approaches to a rational drug discovery paradigm. Curr Top Med Chem.

[6] Ryan CJ, Cimermančič P, Szpiech ZA, Sali A, Hernandez RD, Krogan NJ (2013). High-resolution network biology: connecting sequence with function. Nat Rev Genet.

[7] Tuncbag N, Milani P, Pokorny JL, Johnson H, Sio TT, Dalin S, Iyekegbe DO, White FM, Sarkaria JN, Fraenkel E (2016). Network modeling identifies patient-specific pathways in glioblastoma. Sci Rep.

[8] Kanehisa M, Araki M, Goto S, Hattori M, Hirakawa M, Itoh M, Katayama T, Kawashima S, Okuda S, Tokimatsu T, Yamanishi Y (2008). Kegg for linking genomes to life and the environment. Nucleic Acids Res.

[9] Caspi R, Altman T, Billington R, Dreher K, Foerster H, Fulcher CA, Holland TA, Keseler IM, Kothari A, Kubo A, Krummenacker M, Latendresse M, Mueller LA, Ong Q, Paley S, Subhraveti P, Weaver DS, Weerasinghe D, Zhang P, Karp PD (2014). The metacyc database of metabolic pathways and enzymes and the biocyc collection of pathway/genome databases. Nucleic Acids Res.

[10] Chatr-Aryamontri A, Breitkreutz B-J, Oughtred R, Boucher L, Heinicke S, Chen D, Stark C, Breitkreutz A, Kolas N, O’Donnell L, Reguly T, Nixon J, Ramage L, Winter A, Sellam A, Chang C, Hirschman J, Theesfeld C, Rust J, Livstone MS, Dolinski K, Tyers M (2015). The biogrid interaction database: 2015 update. Nucleic Acids Res.

[11] Orchard S, Ammari M, Aranda B, Breuza L, Briganti L, Broackes-Carter F, Campbell NH, Chavali G, Chen C, del- Toro N, Duesbury M, Dumousseau M, Galeota E, Hinz U, Iannuccelli M, Jagannathan S, Jimenez R, Khadake J, Lagreid A, Licata L, Lovering RC, Meldal B, Melidoni AN, Milagros M, Peluso D, Perfetto L, Porras P, Raghunath A, Ricard-Blum S, Roechert B, Stutz A, Tognolli M, van Roey K, Cesareni G, Hermjakob H (2014). The mintact project–intact as a common curation platform for 11 molecular interaction databases. Nucleic Acids Res.

[12] Schmitt T, Ogris C, Sonnhammer ELL (2014). Funcoup 3.0: database of genome-wide functional coupling networks. Nucleic Acids Res.

[13] Szklarczyk D, Franceschini A, Wyder S, Forslund K, Heller D, Huerta-Cepas J, Simonovic M, Roth A, Santos A, Tsafou KP, Kuhn M, Bork P, Jensen LJ, von Mering C (2015). String v10: protein-protein interaction networks, integrated over the tree of life. Nucleic Acids Res.

[14] Nikitin A, Egorov S, Daraselia N, Mazo I (2003). Pathway studio–the analysis and navigation of molecular networks. Bioinformatics.

[15] Krämer A, Green J, Pollard J, Tugendreich S (2014). Causal analysis approaches in ingenuity pathway analysis. Bioinformatics.

[16] Cowley MJ, Pinese M, Kassahn KS, Waddell N, Pearson JV, Grimmond SM, Biankin AV, Hautaniemi S, Wu J (2012). Pina v2.0: mining interactome modules. Nucleic Acids Res.

[17] Warde-Farley D, Donaldson SL, Comes O, Zuberi K, Badrawi R, Chao P, Franz M, Grouios C, Kazi F, Lopes CT, Maitland A, Mostafavi S, Montojo J, Shao Q, Wright G, Bader GD, Morris Q (2010). The genemania prediction server: biological network integration for gene prioritization and predicting gene function. Nucleic Acids Res.

[18] Wu G, Dawson E, Duong A, Haw R, Stein L (2014). Reactomefiviz: a cytoscape app for pathway and network-based data analysis. F1000Res.

[19] Ivanisenko VA, Saik OV, Ivanisenko NV, Tiys ES, Ivanisenko TV, Demenkov PS, Kolchanov NA (2015). Andsystem: an associative network discovery system for automated literature mining in the field of biology. BMC Syst Biol.

[20] Momynaliev KT, Kashin SV, Chelysheva VV, Selezneva OV, Demina IA, Serebryakova MV, Alexeev D, Ivanisenko VA, Aman E, Govorun VM (2009). Functional divergence of helicobacter pylori related to early gastric cancer. J Proteome Res.

[21] Larina IM, Kolchanov NA, Dobrokhotov IV, Ivanisenko VA, Demenkov PS, Tiys ES, Valeeva OA, Pastushkova LK, Nikolaev EN (2012). Reconstruction of associative protein networks connected with processes of sodium exchange regulation and sodium deposition in healthy volunteers based on urine proteome analysis. Hum Physiol.

[22] Larina IM, Pastushkova LK, Tiys ES, Kireev KS, Kononikhin AS, Starodubtseva NL, Popov IA, Custaud M-A, Dobrokhotov IV, Nikolaev EN, Kolchanov NA, Ivanisenko VA (2015). Permanent proteins in the urine of healthy humans during the mars-500 experiment. J Bioinforma Comput Biol.

[23] Petrovskiy ED, Saik OV, Tiys ES, Lavrik IN, Kolchanov NA, Ivanisenko VA (2015). Prediction of tissue-specific effects of gene knockout on apoptosis in different anatomical structures of human brain. BMC Genomics.

[24] Popik OV, Petrovskiy ED, Mishchenko EL, Lavrik IN, Ivanisenko VA (2016). Mosaic gene network modelling identified new regulatory mechanisms in hcv infection. Virus Res.

[25] Saik OV, Ivanisenko TV, Demenkov PS, Ivanisenko VA (2016). Interactome of the hepatitis c virus: literature mining with andsystem. Virus Res.

[26] Bragina EY, Tiys ES, Rudko AA, Ivanisenko VA, Freidin MB (2016). Novel tuberculosis susceptibility candidate genes revealed by the reconstruction and analysis of associative networks. Infect Genet Evol.

[27] Bragina EY, Tiys ES, Freidin MB, Koneva LA, Demenkov PS, Ivanisenko VA, Kolchanov NA, Puzyrev VP (2014). Insights into pathophysiology of dystropy through the analysis of gene networks: an example of bronchial asthma and tuberculosis. Immunogenetics.

[28] Glotov AS, Tiys ES, Vashukova ES, Pakin VS, Demenkov PS, Saik OV, Ivanisenko TV, Arzhanova ON, Mozgovaya EV, Zainulina MS, Kolchanov NA, Baranov VS, Ivanisenko VA (2015). Molecular association of pathogenetic contributors to pre-eclampsia (pre-eclampsia associome). BMC Syst Biol.

[29] Huang DW, Sherman BT, Lempicki RA (2009). Systematic and integrative analysis of large gene lists using david bioinformatics resources. Nat Protoc.

[30] Maere S, Heymans K, Kuiper M (2005). Bingo: a cytoscape plugin to assess overrepresentation of gene ontology categories in biological networks. Bioinformatics.

[31] Wang J, Zhou X, Zhu J, Gu Y, Zhao W, Zou J, Guo Z (2012). Go-function: deriving biologically relevant functions from statistically significant functions. Brief Bioinform.

[32] Huang DW, Sherman BT, Lempicki RA (2008). Bioinformatics enrichment tools: paths toward the comprehensive functional analysis of large gene lists. Nucleic Acids Res.

[33] de Leeuw CA, Neale BM, Heskes T, Posthuma D (2016). The statistical properties of gene-set analysis. Nat Rev Genet.

[34] Kumar C, Mann M (2009). Bioinformatics analysis of mass spectrometry-based proteomics data sets. FEBS Lett.

[35] Khatri P, Sirota M, Butte AJ (2012). Ten years of pathway analysis: current approaches and outstanding challenges. PLoS Comput Biol.

[36] Alexeyenko A, Lee W, Pernemalm M, Guegan J, Dessen P, Lazar V, Lehtiö J, Pawitan Y (2012). Network enrichment analysis: extension of gene-set enrichment analysis to gene networks. BMC Bioinf.

[37] Glaab E, Baudot A, Krasnogor N, Schneider R, Valencia A (2012). Enrichnet: network-based gene set enrichment analysis. Bioinformatics.

[38] Wei Z, Li H (2007). A markov random field model for network-based analysis of genomic data. Bioinformatics.

[39] Yan J, Risacher SL, Shen L, Saykin AJ. Network approaches to systems biology analysis of complex disease: integrative methods for multi-omics data. Brief Bioinform. 2017:bbx066. 10.1093/bib/bbx066. 10.1093/bib/bbx066 PMC6454489 28679163

[40] Alexeyenko A, Sonnhammer ELL (2009). Global networks of functional coupling in eukaryotes from comprehensive data integration. Genome Res.

[41] Liu M, Fan R, Liu X, Cheng F, Wang J (2015). Pathways and networks-based analysis of candidate genes associated with nicotine addiction. PLoS One.

[42] Sun J, Zhao Z (2010). Functional features, biological pathways, and protein interaction networks of addiction-related genes. Chem Biodivers.

[43] McCormack T, Frings O, Alexeyenko A, Sonnhammer ELL (2013). Statistical assessment of crosstalk enrichment between gene groups in biological networks. PLoS One.

[44] Balasubramanian R, LaFramboise T, Scholtens D, Gentleman R (2004). A graph-theoretic approach to testing associations between disparate sources of functional genomics data. Bioinformatics.

[45] Jensen LJ, Saric J, Bork P (2006). Literature mining for the biologist: from information retrieval to biological discovery. Nat Rev Genet.

[46] Fawcett T (2006). An introduction to roc analysis. Pattern Recogn Lett.

[47] Consortium GO (2015). Gene ontology consortium: going forward. Nucleic Acids Res.

[48] Davies L, Welch HG (2006). Increasing incidence of thyroid cancer in the united states, 1973-2002. JAMA.

[49] Brown LM, Helmke SM, Hunsucker SW, Netea-Maier RT, Chiang SA, Heinz DE, Shroyer KR, Duncan MW, Haugen BR (2006). Quantitative and qualitative differences in protein expression between papillary thyroid carcinoma and normal thyroid tissue. Mol Carcinog.

[50] Giusti L, Iacconi P, Ciregia F, Giannaccini G, Donatini GL, Basolo F, Miccoli P, Pinchera A, Lucacchini A (2008). Fine-needle aspiration of thyroid nodules: proteomic analysis to identify cancer biomarkers. J Proteome Res.

[51] Meier P, Finch A, Evan G (2000). Apoptosis in development. Nature.

[52] Yuan J, Yankner BA (2000). Apoptosis in the nervous system. Nature.

[53] Evan GI, Vousden KH (2001). Proliferation, cell cycle and apoptosis in cancer. Nature.

[54] Ames RM, Macpherson JI, Pinney JW, Lovell SC, Robertson DL (2013). Modular biological function is most effectively captured by combining molecular interaction data types. PLoS One.

[55] Gligorijević V, Janjić V, Pržulj N (2014). Integration of molecular network data reconstructs gene ontology. Bioinformatics.

[56] Rao VR, Lim LE, Fong D, Garga NI, Parko KL (2015). Multicentric castleman’s disease with voltage-gated potassium channel antibody-positive limbic encephalitis: a case report. BMC Neurol.

[57] Vitetta E, Ohara J, Myers CD, Layton J, Krammer P, Paul W (1985). Serological, biochemical, and functional identity of b cell-stimulatory factor 1 and b cell differentiation factor for igg1. J Exp Med.

[58] Bensimon A, Heck AJ, Aebersold R (2012). Mass spectrometry–based proteomics and network biology. Annu Rev Biochem.

[59] Ito T, Chiba T, Ozawa R, Yoshida M, Hattori M, Sakaki Y (2001). A comprehensive two-hybrid analysis to explore the yeast protein interactome. Proc Natl Acad Sci.

[60] Moreau Y, Tranchevent L-C (2012). Computational tools for prioritizing candidate genes: boosting disease gene discovery. Nat Rev Genet.

[61] Zhang Y, Guo GL, Han X, Zhu C, Kilfoy BA, Zhu Y, Boyle P, Zheng T (2008). Do polybrominated diphenyl ethers (pbde) increase the risk of thyroid cancer?. Biosci Hypotheses.

[62] Liz MA, Faro CJ, Saraiva MJ, Sousa MM (2004). Transthyretin, a new cryptic protease. J Biol Chem.

[63] Kim RH, Peters M, Jang Y, Shi W, Pintilie M, Fletcher GC, DeLuca C, Liepa J, Zhou L, Snow B (2005). Dj-1, a novel regulator of the tumor suppressor pten. Cancer Cell.

[64] Chandel NS (2014). Mitochondria as signaling organelles. BMC Biol.

[65] Renault TT, Manon S (2011). Bax: addressed to kill. Biochimie.

[66] Zuckerman V, Wolyniec K, Sionov RV, Haupt S, Haupt Y (2009). Tumour suppression by p53: the importance of apoptosis and cellular senescence. J Pathol.

[67] Levine AJ (1997). p53, the cellular gatekeeper for growth and division. Cell.

[68] Majumder P, Chattopadhyay B, Mazumder A, Das P, Bhattacharyya NP (2006). Induction of apoptosis in cells expressing exogenous hippi, a molecular partner of huntingtin-interacting protein hip1. Neurobiol Dis.

[69] Hartwell LH, Hopfield JJ, Leibler S, Murray AW (1999). From molecular to modular cell biology. Nature.

[70] Rung J, Schlitt T, Brazma A, Freivalds K, Vilo J (2002). Building and analysing genome-wide gene disruption networks. Bioinformatics.

[71] Spirin V, Mirny LA (2003). Protein complexes and functional modules in molecular networks. Proc Natl Acad Sci U S A.

[72] Dutkowski J, Kramer M, Surma MA, Balakrishnan R, Cherry JM, Krogan NJ, Ideker T (2013). A gene ontology inferred from molecular networks. Nat Biotechnol.

[73] Kramer M, Dutkowski J, Yu M, Bafna V, Ideker T (2014). Inferring gene ontologies from pairwise similarity data. Bioinformatics.

